# Cinema audiences reproducibly vary the chemical composition of air during films, by broadcasting scene specific emissions on breath

**DOI:** 10.1038/srep25464

**Published:** 2016-05-10

**Authors:** Jonathan Williams, Christof Stönner, Jörg Wicker, Nicolas Krauter, Bettina Derstroff, Efstratios Bourtsoukidis, Thomas Klüpfel, Stefan Kramer

**Affiliations:** 1 Max Planck Institute for Chemistry, Mainz, Germany; 2 Johannes Gutenberg University of Mainz, Germany

## Abstract

Human beings continuously emit chemicals into the air by breath and through the skin. In order to determine whether these emissions vary predictably in response to audiovisual stimuli, we have continuously monitored carbon dioxide and over one hundred volatile organic compounds in a cinema. It was found that many airborne chemicals in cinema air varied distinctively and reproducibly with time for a particular film, even in different screenings to different audiences. Application of scene labels and advanced data mining methods revealed that specific film events, namely “suspense” or “comedy” caused audiences to change their emission of specific chemicals. These event-type synchronous, broadcasted human chemosignals open the possibility for objective and non-invasive assessment of a human group response to stimuli by continuous measurement of chemicals in air. Such methods can be applied to research fields such as psychology and biology, and be valuable to industries such as film making and advertising.

All living organisms from the smallest plants and bacteria to trees and primates emit chemicals into their local environment^1^,^2^,^3^,^4^. Such chemicals may act as signals, eliciting wide ranging responses^5^,^6^. The atmosphere has been shown to be an effective conduit for chemical communication between plants and plants^7^, plants and insects^8^, insects and insects^9^. Yet the extent, or even existence of airborne chemical communication between humans remains controversial^10^,^11^. Despite reported chemosignal volatiles in human tears affecting testosterone levels^12^, armpit and sweat odours interpreted as fear signals^13^,^14^,^15^, sleeping babies responding to lactating breast volatiles^16^,^17^,^18^ and menstrual synchronization^19^, no human pheromone (an evolved chemical signal between humans) has been reliably and reproducibly identified^20^. Generally, studies reported to date have been small in scale (number of people and measurements), subjectively assessed^13^,^14^, and often with unnaturally high concentrations of bioassays, due to the analytical methods available. To screen groups of people for potential emotion signaling molecules at natural levels we have conducted a largescale study involving more than 9500 cinemagoers who viewed 108 screenings of 16 different films (including comedy, horror and romance, see Table 1a). During the films, audiences were subjected to audiovisual stimuli while outside air was directed into the cinema through floor vents and out through ceiling vents (normal operating practice), and in the outflow, the concentration of over 100 trace gas species was measured using proton transfer reaction mass spectrometry(PTR-MS) and infra-red spectroscopy. Data was collected at 30 second time resolution and with sub-ppb(10^−9^) detection limits to investigate potential causal links between the audiovisual stimuli and audience emitted chemicals.

Of the 872 volatile compounds identified in human breath^1^, a fraction is thought to be produced endogenously. These compounds can be used to track chemical changes within the body, over long (with age)^21^,^22^ and short timescales (medication response, food, disease or exercise)^2^,^23^,^24^,^25^. Within this cinema based study we hypothesize that if films elicit strong emotional responses then volatile products from the internal biochemical response (cardiovascular, skeletomuscular, neuroendocrine, and autonomic nervous system)^26^,^27^ may be vented shortly afterwards over the lungs, and observed as transient peaks in concentration in air exiting the cinema. Full details of the experimental set-up and instrumentation is given in the method section.

## Results

**Figure 1:**
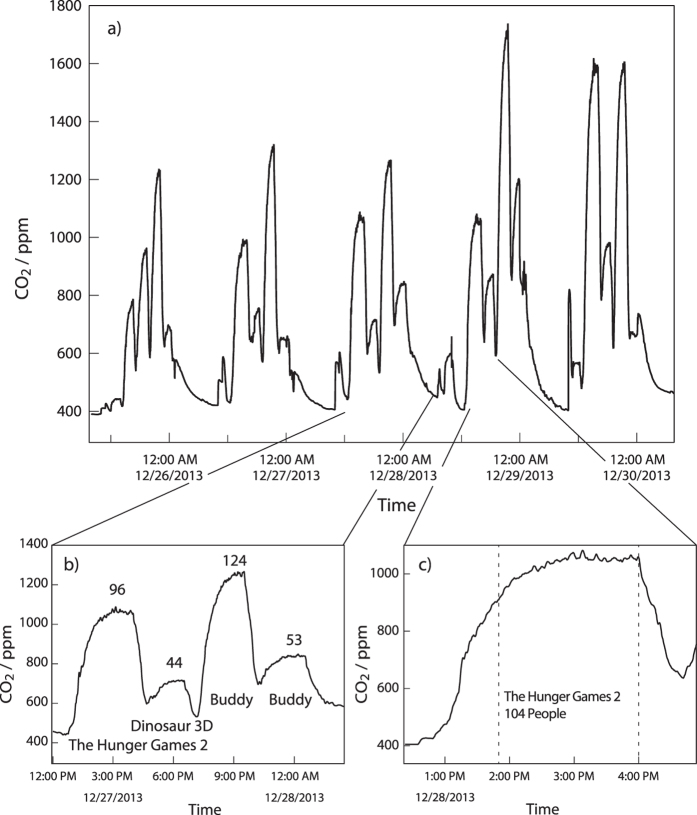
Selected sections of the CO_2_ measurements, (**a**) 5 days, (**b**) 1 day and (**c**) 1 film. The numbers above the peaks indicate the number of people in the audience.

Figure 1 shows sections of the CO_2_ data measured in air from the Mainz Cinestar cinema. In Fig. 1a, large CO_2_ peaks can be observed between 26^th^ and 30^th^ December, each corresponding to the screening of a particular film. Prior to a film starting in the empty cinema, CO_2_ approximates to background levels (ca. 400 ppm) as ambient air is continually drawn through the cinema from outside. People exhale air with circa 4% CO_2_, so that as the audience arrives, CO_2_ levels increase, rapidly at first, and then more slowly as the equilibrium value is approached after about ninety minutes, reaching levels between 1000–2400 ppm. This is some 2 to 8 times the current ambient background levels (400 ppm), but well below the European indoor standard limit of 3500 ppm. In effect, the cinema is a small scale analogue of the on-going planetary scale increases in CO_2_ in which additional anthropogenic CO_2_ sources from fossil fuel usage must equilibrate with the slow uptake rates into the ocean, vegetation and soils^28^. At the end of each film the CO_2_ level falls abruptly as the audience departs, generating a “shark-fin” profile for CO_2_.

**Figure 2:**
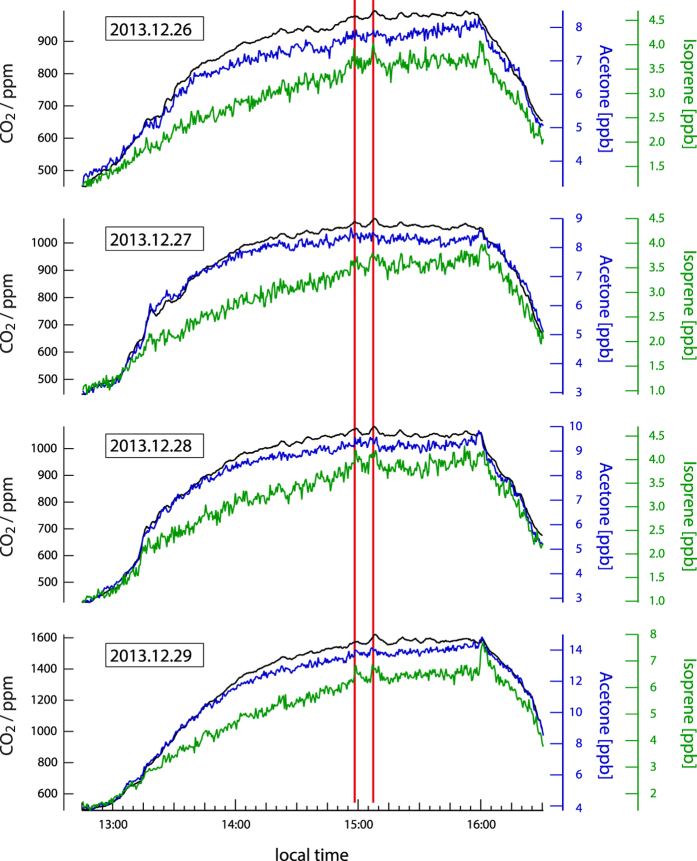
Measurements of CO_2_, isoprene and acetone taken during four separate screenings of “Hunger Games 2”.

Figure 1b shows CO_2_ measurements and audience numbers for a day on which four films were screened, “Hunger Games 2”, “Dinosaurs 3D” and “Buddy” twice. Those films with higher attendance have correspondingly higher CO_2_. Figure 1c displays the CO_2_ profile of a single film, “Hunger Games 2”. Clearly the CO_2_ trace does not increase smoothly with time, as would be expected from a constant emission source, but rather small peaks are discernable despite the cinema ventilation rate remaining constant. These CO_2_ peaks would be generated if the audience’s pulse and breathing rate were momentarily increased in response to scenes in the film. Figure 2 shows measurements from four showings of “Hunger games 2” on sequential days between December 26^th^–29^th^ with attendances of 87, 96, 104, and 186 people respectively. Two distinct peaks in CO_2_ occurring around 15:00, highlighted by the red vertical lines, are visible on all days, indicating that the physiological response induced in each audience is reproducible. The pattern of CO_2_ peaks shown in Fig. 1c was characteristic of the film “Hunger Games 2” and in many cases it was possible to identify the different films from the CO_2_ profile by eye.

The mixing ratios of isoprene (C_5_H_8_) and acetone (C_3_H_6_O), which are among the most abundant exhaled organic trace gases^1^,^2^, are shown with CO_2_ for four film screenings in Fig. 2. Acetone is a soluble gas (in blood and water) that has been linked to fat catabolism, while isoprene is an insoluble gas linked to cholesterol synthesis^2^,^23^. In Fig. 2 peaks can be seen in the isoprene trace and to a lesser extent for acetone, although acetone mixing ratios were twice as high. Isoprene levels in the cinema are similar to levels reported from aircraft flying low over the pristine Amazon rainforest (1–3 ppb)^29^ while acetone levels generated by the audience (~8 ppb) are approximately twice that found in forested environments^30^ and city air^31^. The two distinct peaks around 15:00 previously noted in CO_2_ are also visible in isoprene, and additionally a further large isoprene peak is observed at the end of each film (16:00). Breath analyses of individuals on an ergometer have shown that isoprene can be stored in muscle tissue, and that limb movement increases isoprene in breath^25^. The mass exodus of people at the end of the film is therefore the likely cause of the isoprene peak at 16:00 coincident with rapidly falling CO_2_. However, the two other outstanding peaks in isoprene appear during the film when the audience is seated (15:00 and 15:10). These times correspond to key moments in the film when the heroine’s dress catches fire and when the final battle begins. Previous studies have indicated that breath holding^32^ and twitching muscles^25^ could potentially enhance isoprene emission over acetone. Another possibility is that isoprene is linked to cortisol production via cholesterol. Whatever the mechanism behind the release, the peaks in isoprene were reproduced in all four screenings of the film at the same time, meaning that each set of cinemagoers broadcasted chemicals into the air in synchrony to on-screen events.

**Figure 3:**
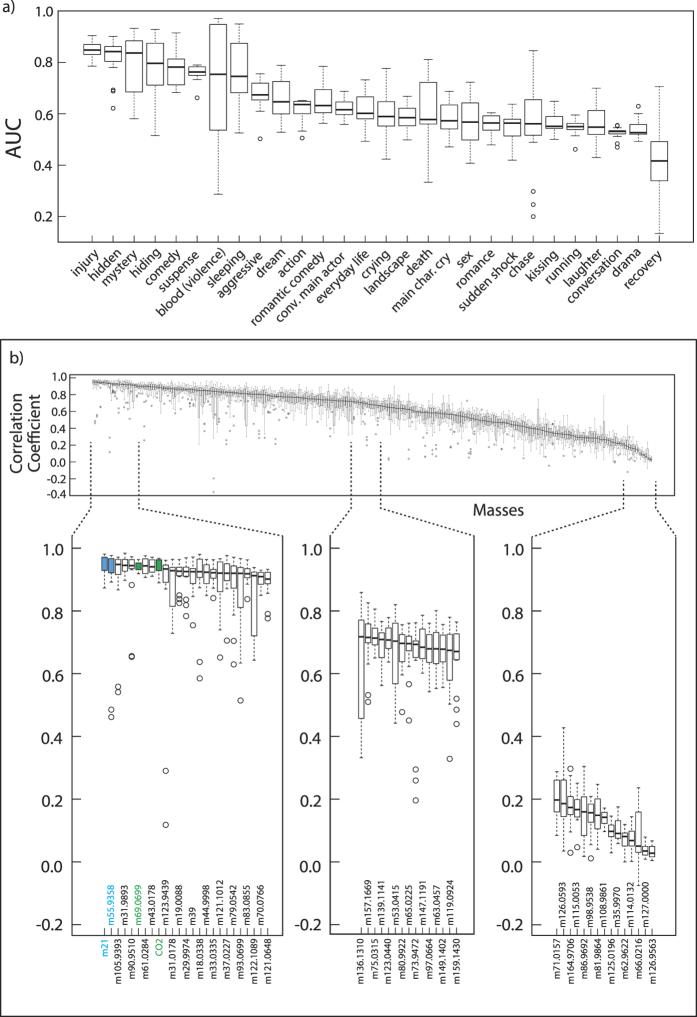
Shown are the results when two thirds of the whole film screening dataset is randomly selected (15 times) and the resultant model tested on the remaining third. The boxes indicate the extent of 25% of the data either side of the median (solid line). The dashed vertical line represents the lowest/highest datapoints that are still in the 1.5 interquartile range while the circles are outliers. (**a**) shows AUC which expresses the ratio between true positives (when the model correctly predicted labels based on mass decision trees) and false positives (backward prediction). A random prediction produces an AUC value of 0.5. (**b**) shows the ability of an individual mass to be predicted by the labels (forward prediction). The performance of this prediction versus the real value for VOC mixing ratios is given as the Pearson’s correlation coefficient (r). High correlation coefficients indicate the predictive model was successful for that particular species, and not that all species with high correlation coefficients are inter-correlated.

To determine whether causal links exist between levels of all chemicals measured and events in the film, it was necessary to annotate the films with scene content labels. A set of scene labels (Table 1b,c) was defined based on genres in the IMDb database (e.g. comedy), on objective subheadings (e.g. chase) and psychological studies (happy to sad and excited to calm). These labels were applied to the films by ten individuals independently (see method for details). All data were then statistically normalized and random forests were constructed for each mass and CO_2_, for each 30 second timestep within a 10 minute window, and for each label^33^. Each random forest based model was generated based on a randomly selected subset of two thirds of the data and then evaluated on the remaining third. This procedure was then repeated 15 times, using the Mainz Mogon supercomputer. A set of models were trained in a process called backward prediction to determine how well the present label was predicted by the future mass (in the next 5 minute time window). Figure 3a shows film scene labels plotted against AUC (Area Under Curve, see method) which expresses the ratio between true positives (when the model correctly predicted labels based mass decision trees) and false positives. A random prediction produces an AUC value of 0.5. Many of the labels showed a significant relationship with measured masses. The highest AUCs observed were for the labels “injury” (0.85), “hidden” (0.83), “mystery” (0.81) and “hiding” (0.79), all of which were subcategories of the label “suspense” which itself showed an AUC value of 0.75. The label comedy was also predictable based on the measured chemicals (AUC = 0.78). In contrast, the label “chase” (AUC = 0.55) could not be predicted by the model.

In parallel we investigated the ability of an individual mass to be predicted by the labels (forward prediction). The performance of this prediction versus the measured mixing ratio is given as the Pearson’s correlation coefficient (r) in Fig. 3b. Strong correlation was found between model predicted and measured CO_2_, as well as for the predicted and measured water sensitive reagent clusters m21 and m39. Both water and CO_2_ are introduced to the cinema primarily by breath. Among the best correlated masses was isoprene (r = 0.91), which is presented qualitatively for the film “Hunger Games 2” in Fig. 2. Some masses with high correlations have not been observed or identified in previous studies (e.g. 105.93, r = 0.92) while other masses exhibit no significant correlation.

Table 2 shows the best correlated masses and labels based on backward prediction. A filter of AUC >0.5 and significance level <0.05 was applied to all data. “Significance” here is the result of a statistical T-test (between an evaluation based on all masses and an evaluation with one mass omitted, this mass is given in Table 2). Therefore higher AUC and lower significance values indicate stronger potentially causal links. The labels with the highest overall causal link to the measured species were “injury” and “comedy”. Among the chemicals linked to injury scenes are methanol (mass 33.0335), acetaldehyde (mass 45.0335), 2-furanone (mass 85.0284), and butadiene (mass 55.0580). These compounds have all been previously detected in human breath^1^. Although the masses 100.9380 and m73.9472 were also significantly linked, no plausible identification could be made based on combinations of C, H, and O. Curiously, the mass 374.08 also shows a causal link to injury scenes despite being associated with polysiloxane which is found in cosmetics and conditioning shampoo. This may be related to emotionally induced body temperature variations rather than to breath. The film labels “chase” and “romance” both did not show significant causal links with any measured masses.

## Discussion

Interestingly, the two film scene labels with the most significant linkage to chemicals measured were “suspense” and “comedy”. These could be interpreted as an evolutionarily advantageous alert/stand-down signal, if perceivable by others^34^. Humans possess a very well developed sense of smell^35^, and new evidence suggests that recall is more effective^36^, and our perception of faces changes with odours present^37^. Therefore the chemical accompaniment generated by the audience has the potential to alter the viewer’s perception of a film.

There are several important consequences of our finding that human beings respond to audiovisual cues through breath emissions. Firstly, in the field of medicinal breath analysis, where chemical markers for diseases such as cancer are being sought^2^, emotionally induced emissions have the potential to confound disease marker identification. The strong response found here for “suspense” suggests that a patient’s state of anxiety should be taken into account in future medicinal breath studies. These findings also have obvious industrial applications where an objective assessment of audiovisual material is sought from groups of people, for example, in advertising, video game design or in film making.

## Method

### Cinema/Movie Theater

**Figure 4:**
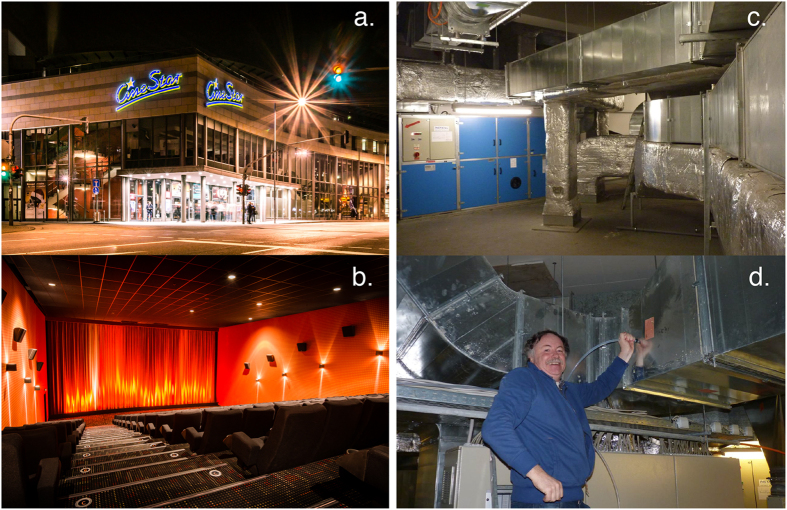
(**a**) The Cinestar Cinema in Mainz, Germany, (**b**) The 230 seat capacity cinema audioreum, (**c**) the air ventilation system, (**d**) insertion of the Teflon inlet into the 75 × 75 cm ventilation system. (**a,b**) are reproduced with permission from Cinestar.

All data were recorded at the Cinestar Cinema complex in Mainz (Fig. 4a), Germany between 1^st^ December 2013 and 14^th^ January 2014. Of the 14 screen multiplex, two separate screen rooms were used (see Fig. 4b, Cinema 2 capacity 230, and Cinema 7 capacity 230). During a film the entrance doors were closed and ambient air was circulated from outside into the room through vents under the banked seating and out via ceiling mounted openings so that the screening room was flushed entirely circa 6 times per hour. The measurement instruments (PTR-ToFMS and the CO_2_ detector, see below for details) were located outside the screening room (to avoid possible noise disturbance), in a technical room that contained the outgoing air vents (75 × 75 cm square stainless steel) and associated control systems for all auditoriums, see Fig. 4c. An inlet was inserted into the midpoint of the exit flow vent and a 10 L/min flow was drawn through ¼” OD (0.625 cm) Teflon line continuously, see Fig. 4d. The films viewed and the number of screenings are given in Table 1a. This is a study of ambient air and the chemical changes within it caused by entirely anonymous groups of people in a public space. No personal data concerning the cinemagoers was collected, no individuals identified, only the number of people present were recorded by way of the ticket sales.

### Proton Transfer Reaction Time of Flight Mass Spectrometer

Volatile Organic Compounds (VOC) were measured using a commercial PTR-ToFMS (Proton Transfer Reaction Time of Flight Mass Spectrometer, PTR-ToF-MS-8000, Ionicon Analytik GmbH, Innsbruck, Austria)^38^,^39^. The measurement technique is based on the low pressure (ca. 2 mbar) protonation of molecules with a proton affinity higher than water by H_3_O^+^ ions (691 kJ mol^−1^) that are generated in a hollow cathode discharge chamber flushed with water vapour. All protonated molecular ions are accelerated by an electrical field to the same kinetic energy such that the resultant velocity of the ions depends on the mass-to-charge ratio. Hence, the time-of-flight is used to measure the velocity, from which the mass-to-charge ratio can be determined. The TOF was configured in the standard V mode with a mass resolution of approximately 3700 m/∆m. Mass spectra were collected ranging from m/z 10–400 with a TOF acquisition sampling time per channel of 0.1 ns. The instrument was operated with a drift pressure of 2.20 hPa (E/N 137 Td) and a drift voltage of 600 V. For mass calibration, 1,3,5-trichlorobenzene was used as an internal standard by permeating 1,3,5-trichlorobenzene into a 1 mm section of 1/8” (1.58 mm) Teflon tubing used in the inlet system. Data post-processing and analysis was performed by using the program “PTR-TOF DATA ANALYZER”, which is described elsewhere^40^. The PTR-ToFMS was calibrated with a commercial pressurized gas standard mixture (Apel-Riemer Environmental Inc., Broomfield, USA) of known mixing ratio. The overall uncertainty was 15%. The calculated detection limit (3σ of the noise) of identified masses was between15 ppt and 155 ppt. Signals were normalized to H_3_O^+^ ions and the first water cluster H_3_O(H_2_O)^+^ by means of the following formula:


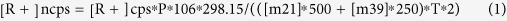


here [R+]ncps is the normalized counts per second, [R+] is the reagent ion, P the pressure, T the temperature, [m21] the counts per second of the O^18^ isotope of H_3_O^+^ and [m39] the counts per second of the ^18^O isotope of the first water cluster of the primary ion. The signal is normalized to a temperature of 298.15 K and a pressure of 2 mbar. The humidity dependence of the PTR-ToF-MS sensitivity was tested for a suite of compounds including key breath species such as isoprene and acetone shown in Fig. 2. The sensitivity was weak, varying in the order of 3% for the ambient conditions in the cinema and therefore we can exclude humidity dependent variations in sensitivity as the cause of the peaks shown.

### Carbon Dioxide (CO_2_) measurement

CO_2_ was measured at 1 Hz using a commercially available Li-COR Li-7000 system. The Li-7000 monitor was calibrated using a standard containing 509 ± 10 ppmv of CO_2 _ppmv (Air Liquide, Germany) before and during the campaign. The instrument specifications state that the response is linear up to 3000 ppmv. Post campaign the linearity of the response was confirmed to 3400 ppmv using a second standard gas (10% CO_2_, Air Liquide, Germany).

### Film scene annotation

In order to assess the data for relationships between film scene content and trace gas behavior it was necessary to annotate the film scene content at high time resolution, from a set of preselected labels. Although several approaches to film scene annotation have been reported, including scene change frequency and both audio and visual cues^40^,^41^,^42^,^43^, as yet no standardized procedure exists. Suitable independently derived time resolved annotations were also not available from film censor boards nor from the subsequently published film DVDs. Instead, ten volunteers individually viewed the films and allocated descriptor annotations as a function of the film duration using a custom made interface. Each film was labelled at least five separate times. Three different types of scene labels were used. The first set was general in nature and described the film genre using terms from the Internet Movie Database (IMDb). These included terms such as “comedy”, “suspense” or “romantic.” The second set was more specific and referred directly to the scene content such as “chase”, “laughter” or “kiss”, “house pet” or “injury”. These terms were kept deliberately objective to minimize potential labelling differences between individuals caused by personal perception. Finally, we have adopted an emotional assessment scheme that has been previously used by psychologists^44^. It consists of two separate five point scales, one ranging from happy to sad and the other from excited to calm. The labels produced by the individual volunteers were then averaged and used only when two thirds of the individuals agreed. The labels were created to match the datapoint frequency (1 every 30 seconds). A full list of scene labels is in Table 1b,c and a comprehensive description of all data mining approaches applied to the dataset given by Wicker *et al.*^33^.

### Data Mining

This study was designed to determine whether causal links exist between levels of volatile organic compounds and CO_2_ emitted in a cinema auditorium and events in the film. While it is easy to examine the variance with time of a single molecular species for a single film by simple graphical methods (see for example Figs 1 and 2), to analyze the entire suite of measured masses (including unidentified mass species) at thirty second intervals with all the labels from all the films for causal relationships and possible interdependencies requires a more sophisticated and systematic data mining approach. Full details of the data mining algorithms applied are given by Wicker *et al.*^33^, however, the generalized approach is summarized below. Data mining algorithms were applied to analyze the VOC and label data within a 10 minute window around a given measurement datapoint (5 minutes backwards and 5 minutes forwards). The first method applied was forward prediction, whereby the VOC mixing ratios are predicted based on regression from past VOC mixing ratios and the film labels. The second method was termed backward prediction, as it used VOC changes ahead of a given point in time to predict the current associated label. In order to evaluate the coherence of the two types of models, the forward prediction model and the backward prediction model, we used the predictions of the forward prediction model as an input to the backward prediction model and compared the resulting predicted values with the actual values. The overall product of the backward prediction are tables of VOC signal intensities (measured as mass-to-charge ratios in the mass spectrometer) that are associated with a given label and the error in the prediction expressed as the area under the receiver operating characteristic (ROC) curve (AUC, sometimes also called AUROC, see Table 2) and a significance. The AUC expresses how well a classifier (in this case the label) ranks the cases of one class before those of another class (in our case: those of one scene label before those of all others). An AUC value of 1 would mean that the label was predicted perfectly from mass signals, while a value of 0.5 indicates that the predictive performance was equivalent to a random selection^45^. The p-value results from a statistical test that compares the performance of a machine learning model using all masses as input to the performance of a model using all but one mass as input. The difference between these two cases is tested using a corrected paired t-test^46^. The t-test returns a significance measure in terms of p-values, the lower the p-value, the more probable is a relationship between the left out mass and the target label. Whereas in most cases, an adjustment like Holm-Bonferoni should be performed on the tests, this is not necessary in this case, as we only searched for indications for further analysis, which we also can get from uncorrected values. The results of the two (significance level and AUC in Table 2) expresses the significance of the relationship with low number of p-values and high numbers of AUCs indicating higher degrees of dependence.

## Additional Information

**How to cite this article**: Williams, J. *et al.* Cinema audiences reproducibly vary the chemical composition of air during films, by broadcasting scene specific emissions on breath. *Sci. Rep.* **6**, 25464; doi: 10.1038/srep25464 (2016).

## Figures and Tables

**Table 1:** 

(a)		
Film	Number of Screenings	
Buddy	17	
Walking with Dinosaurs	15	
The Hobbit – The desolation of Smaug	15	
The Secret Life of Walter Mitty	15	
The Hunger Games 2	10	
Carrie	7	
Suck me Shakespeare	5	
The Little Ghost	4	
Journey to the Christmas Star	4	
Paranormal Activity 6	4	
Belle and Sebastian	3	
The Counselor	3	
Machete Kills	2	
Cloudy with a chance of Meatballs 2	2	
The Physician	1	
Bolshoi: Sleeping Beauty	1	
**Total**	**108**	
**(b)**		
**Content Labels**		
Label	Sub-label	Relative Frequency
Everyday Life		0.025
Dream		0.016
Landscape		0.040
Conversation		0.680
	Aggressive	0.008
	Conv. Main Actor	0.321
Action		0.141
Death		0.022
Running		0.031
Recovery		0.001
Laughter		0.004
Sleeping		0.001
Blood (violence)		0.028
Sex		0.003
Kissing		0.009
Crying		0.007
	Main Char. Cry	0.006
Injury		0.008
Sudden shock		0.026
**(c)**		
**Genre Labels**		
Suspense		0.283
	Chase	0.002
	Hidden Threat	0.005
	Hiding	0.002
Comedy		0.054
Romantic Comedy		0.002
Mystery		0.002
Romance		0.014
Drama		0.019

A summary of the film screenings (a), labels and associated sublabels used in the film scene content (b) and film scene genre (c) annotation.

**Table 2:** Film labels and masses with significant causal links are shown (Injury, Comedy, and Mystery) and two examples where masses and labels were not linked (Romance and Chase).

Injury				
Mass	Sig	AUC	Formula	Possible ID/Comment
none		0.84929		
m 374.082	0.004	0.81808		siloxanes
m 73.947	0.015	0.82840		
m 85.028	0.018	0.82524	C4H4O2	2 (5H)-furanone (Br)
m 105.034	0.030	0.81353	C4H8OS	3-(methylthio)-propanal (F, U, M)
m 100.938	0.031	0.81562		
m 40.974	0.035	0.82323		
m 45.034	0.040	0.82347		Acetaldehyde (F, U, Br, Sk, M, Bl, Sa), Ethylene oxide (F, Br)
m 55.058	0.040	0.83322		Butadiene (Br), Butyne (Br)
m 33.034	0.044	0.82877		Methanol (F, Br, M, Bl)
Comedy				
none		0.77843		
m 235.208	0.010	0.75878	C15H26N2	
m 111.080	0.031	0.76360	C7H10O	1,3-cyclohexadien-1-yl methyl ether (Br), 2-ethyl-5-methylfuran (F, U, Br), (E, Z)-2,4-heptadienal (M), 3-methyl-2-cyclohexen-1-one (U, Br), propylfuran (Br), 2,3,5-trimethylfuran (U, Br)
m121.065	0.045	0.76266	C8H8O	Acetophenone (F, U, Br, Sk, M, Sa), 2,3-dihydro-1-benzofuran (Br), 4-methylbenzaldehyde (F), phenyl acetaldehyde/phenylethanal/benzene acetaldehyde (F, M)
Mystery				
none		0.79193		
m 217.204	0.024	0.73270	C15H20O	a-hexyl cinnamaldehyde (Sk)
m 108.959	0.030	0.69253		
m 159.143	0.040	0.69335	C9H18O2	1-methylhexyl acetate (Sk), isoamyl butanoate (Br), heptanoic acid, ethyl ester (F), hexanoic acid, propyl ester (F), 3-methylbutanoic acid, butyl ester (F), 2-methyloctanoic acid (Sk), 2-methylbutyl 2-methylpropanoate (Br), nonanoic acid (U, Br, Sk, M, Sa), pentanoic acid, butyl ester (F), propanoic acid, hexyl ester (F)
m 138.140	0.046	0.71237	13CC9H16	Isotope of monoterpenes
Romance				
none		0.55738		
m 95.049	0.157	0.54349	C6H6O	
m 79.002	0.165	0.54388		
m 70.077	0.289	0.54591	13CC4H8	Isotope of isoprene
Chase				
none		0.55248		
m122.109	0.128	0.47477	C8H11N	
m100.084	0.135	0.47568	C5H9NO	
m164.971	0.169	0.47155		
m135.030	0.175	0.50660	C8H6O2	

The AUC value for “none” means the result with the complete dataset, and the values below are AUCs when the stated mass is removed from the model. Significance and AUC are given for each mass as well as an elemental formula and possible molecular identities based on previous measurements from human emissions summarized by de Lacy Costello *et al.*^1^. The abbreviations refer to where the species were previously measured Br = Breath, Sk = Skin, U = Urine, F = Faeces, Bl = Blood and M = Mucus).
